# Novel Affibody Molecules Targeting the HPV16 E6 Oncoprotein Inhibited the Proliferation of Cervical Cancer Cells

**DOI:** 10.3389/fcell.2021.677867

**Published:** 2021-05-24

**Authors:** Jinshun Zhu, Saidu Kamara, Qi Wang, Yanru Guo, Qingfeng Li, Linlin Wang, Jingjing Chen, Qianqian Du, Wangqi Du, Shao Chen, Shanli Zhu, Jun Chen, Maoping Chu, Lifang Zhang

**Affiliations:** ^1^Department of Microbiology and Immunology, Institute of Molecular Virology and Immunology, School of Basic Medical Sciences, Wenzhou Medical University, Wenzhou, China; ^2^Children’s Heart Center, Institute of Cardiovascular Development and Translational Medicine, The Second Affiliated Hospital and Yuying Children’s Hospital of Wenzhou Medical University, Wenzhou, China

**Keywords:** HPV, oncoprotein E6, affibody molecules, cervical cancer, molecular imaging, targeted therapy

## Abstract

Despite prophylactic vaccination campaigns, high-risk human papillomavirus (HPV)-induced cervical cancer remains a significant health threat among women, especially in developing countries. The initial occurrence and consequent progression of this cancer type primarily rely on, E6 and E7, two key viral oncogenes expressed constitutively, inducing carcinogenesis. Thus, E6/E7 have been proposed as ideal targets for HPV-related cancer diagnosis and treatment. In this study, three novel HPV16 E6-binding affibody molecules (Z_HPV16E6_1115, Z_HPV16E6_1171, and Z_HPV16E6_1235) were isolated from a randomized phage display library and cloned for bacterial production. These affibody molecules showed high binding affinity and specificity for recombinant and native HPV16 E6 as determined by surface plasmon resonance, indirect immunofluorescence, immunohistochemistry, and near-infrared small animal optical imaging *in vitro* and *in vivo*. Moreover, by binding to HPV16 E6 protein, Z_HPV16E6_1235 blocked E6-mediated p53 degradation, which increased the expression of some key p53 target genes, including BAX, PUMA and p21, and thereby selectively reduced the viability and proliferation of HPV16-positive cells. Importantly, Z_HPV16E6_1235 was applied in combination with HPV16 E7-binding affibody Z_HPV16E7_384 to simultaneously target the HPV16 E6/E7 oncoproteins, and this combination inhibited cell proliferation more potently than either modality alone. Mechanistic studies revealed that the synergistic antiproliferative activity depends primarily on the induction of cell apoptosis and senescence but not cell cycle arrest. Our findings provide strong evidence that three novel HPV16 E6-binding affibody molecules could form a novel basis for the development of rational strategies for molecular imaging and targeted therapy in HPV16-positive preneoplastic and neoplastic lesions.

## Introduction

Cervical cancer is the second most common cause of cancer-related mortality among females worldwide, with approximately 570,000 new cases and 311,000 deaths annually (Serrano et al., 2018). Compelling evidence suggests that persistent infection with high-risk human papillomaviruses (HR HPVs), including HPV 16, 18, 31, 33, 35, 39, 45, 51, 52, 56, 58, and 59 is the major etiological agent in cervical carcinogenesis (zur Hausen and de Villiers, 1994). Among these, HPV16 and HPV18 are the most prevalent genotypes, accounting for approximately 62.6 and 15.7% of cervical neoplasias, respectively (Walboomers et al., 1999; Tommasino, 2014). Concurrent chemoradiation is the main treatment for locally advanced cervical cancer, and this approach yields a 5-year disease-free survival rate of 65 to 78%, indicating that there is still ample room for improvement (Cohen et al., 2019). Diagnostically, HPV DNA tests are very sensitive for the diagnosis of HPV infection; however, their specificity is limited for cervical precancer and early cancers, as they detect the many benign HPV infections in addition to the less frequent, clinically important infections linked to disease (Simon et al., 2011; Schmitt et al., 2013). Therefore, the development of diagnostic and treatment strategies is urgently required to improve the diagnosis and clinical outcomes of patients with cervical cancer.

The HPV viral genome contains 6 “early” (E1, E2, E4, E5, E6, and E7) genes and 2 “late” (L1 and L2) genes and is actively transcribed in infected cells (Estêvão et al., 2019). For persistent infections, the episomal viral genome integrates into the host chromosome, leading to invariably retained and expressed E6 and E7 oncoproteins. In contrast to E6, E7 plays a crucial role in the early stage of carcinogenesis by stimulating proliferation. E6 protein primarily promotes malignant progression: recruitment of a cellular ubiquitin ligase (E6AP) and degradation of tumor suppressor p53 overcomes cell cycle arrest and/or apoptosis, allowing increased DNA damage, and induction of telomerase contributes to immortalization and cancer development. HPV16 E6 and E7 oncoproteins also target multiple signaling proteins by perturbing several signaling pathways that are vital for malignant transformation and for retaining the malignant phenotype of cervical cancers (Tan et al., 2012; Almeida et al., 2019; Estêvão et al., 2019; Pal and Kundu, 2020). Indeed, interference with HPV16 E6/E7 activity by siRNA (Jung et al., 2015, 2012) or small-molecule inhibitor (Dymalla et al., 2009; Celegato et al., 2020) has been found to exert strong antioncogenic effects on HPV16-positive cancer cells *in vitro* and *in vivo*. In addition, it is worth noting that HPV16 E6 and E7, as two non-cellular oncoproteins, are not expressed in normal cells (Dymalla et al., 2009). Therefore, HPV16 E6 and E7 oncoproteins are the ideal targets for molecular diagnosis and targeted therapy of HPV16-related malignancies.

Affibody molecules, a newly emerging class of affinity proteins based on scaffolds other than the immunoglobulin fold, are derived from one of the IgG-binding domains of staphylococcal protein A (Nilsson et al., 1987). By randomly mutating thirteen specific amino acid residues of the three α-helix regions, large affibody libraries can be constructed, from which potent binders for theoretically any desired target molecules can be selected using various display technologies, e.g., phage display technology (Löfblom et al., 2010; Gebauer and Skerra, 2020). With the simple, robust structure addition to small molecular size (58 amino acids, 6.5 kDa) and cost-efficient production, affibody molecules are widely applied, for example, as *in vivo* molecular imaging reagents and to block receptor signals (Ståhl et al., 2017; Tolmachev and Orlova, 2020). To date, over 500 studies have been published in which affibody molecules targeting approximately 50 different proteins have been isolated and serve as high-affinity ligands in a variety of applications^1^. The affibody-targeted proteins, including HER2 (human epidermal growth factor receptor 2; Orlova et al., 2006), EGFR (epidermal growth factor receptor; Wu et al., 2020), TNF-α (tumor necrosis factor-α; Löfdahl et al., 2009), and transcription factor c-Jun (Lundberg et al., 2009), as well as EBV LMP2 (Epstein-Barr virus latent membrane protein 2; Zhu J. et al., 2020; Zhu S. et al., 2020) and HPV16 E7 (human papillomavirus type 16 E7; Xue et al., 2016; Jiang et al., 2018), which have been reported by our research team.

Herein, we reported the selection and characterization of three HPV16 E6-binding affibody molecules (Z_HPV16E6_ affibodies) for their target binding ability to recombinant and native HPV16 E6 protein *in vitro* and their usage in molecular imaging in tumor-bearing mice. Further investigations showed that by binding to HPV16 E6, affibody Z_HPV16E6_1235 blocked E6-mediated p53 degradation and specifically inhibited the cell viability and proliferation of HPV16-positive cancer cells. Moreover, we also showed that the combination of Z_HPV16E6_1235 with Z_HPV16E7_384 to simultaneously target the HPV16 E6 and E7 oncoproteins had a greater efficacy than either modality alone. Mechanistically, our data revealed that the synergistic antiproliferative activity primarily depends on the induction of cell apoptosis and senescence but is not related to cell cycle arrest. To our knowledge, this is the first report of HPV16 E6-binding affibody molecules as novel probes for the *in vivo* imaging diagnosis of HPV16-positive tumors. Most importantly, our study provides the first evidence that simultaneous targeting of HPV16 E6 and E7 with affibodies Z_HPV16E6_1235 and Z_HPV16E7_384 can significantly enhance antiproliferative activity in HPV16-positive cancer cells.

## Materials and Methods

### Animals, Cells and Vectors

Female BALB/c-nude mice, 4 to 6 weeks old, were purchased from Shanghai SLAC laboratory animal CO., LTD (Shanghai, China), and kept at the animal facility of Wenzhou Medical University, China under specific pathogen-free (SPF) conditions. The near-infrared (NIR) small animal optical imaging experiment was approved by the Ethical Committee of Wenzhou Medical University. Murine HPV16-positive TC-1 cells, obtained from primary epithelial cells of C57BL/6 mice cotransformed with c-Ha-ras and HPV16 E6/E7 oncogenes, were kindly offered by Xuemei Xu (Chinese Academy of Medical Sciences and Peking Union Medical College, Beijing, China). Human HPV-positive CaSki (HPV16), HeLa229 (HPV18 cell line, applied as HPV16 negative control) cells and nasopharyngeal carcinoma C666-1 cells (HPV negative control cell line) were bought from the ATCC (American Type Culture Collection) and cultured according to the supplier’s instructions. The *Escherichia coli BL21(DE3)* and *p*ET21a(+) vector were purchased from ATCC and Novagen, respectively.

### Selection of Affibody Ligands

In our previous study, a 14-kDa high-purity HPV16 E6 recombinant protein was prepared (Zhang et al., 2020) and used as a target protein during selection. Screening for potential HPV16 E6-binding affibodies was conducted following protocols established in our laboratory, which are described in detail in several previous studies (Xue et al., 2016; Zhu J. et al., 2020; Zhu S. et al., 2020). After pressure biopanning, phage-based ELISA screening and DNA sequencing, the sequences derived from inserted fragments in selected phage clones were potential affibody molecules with elevated affinity for HPV16 E6.

### Subcloning and Production of Affibody Molecules

The genes encoding selected affibody molecules were subcloned into the *Nde*I and *Xho*I sites of *p*ET21a(+), generating affibody constructs in fusion with a *C*-terminal 6 × His-tag. The resulting plasmids encoding each affibody molecule were prepared and transformed into E. *coli BL21(DE3)*. After 6 h of isopropyl β-D-1-thiogalactopyranoside (IPTG, Sigma-Aldrich Co., St. Louis, MO, United States) induction, the proteins were purified with Ni-nitrilotriacetic acid (NTA) agarose resin according to the manufacturer’s recommendations (Qiagen, Hilden, Germany). Purified proteins with the correct molecular mass were confirmed by SDS-PAGE and a Western blotting assay using an anti-His-tag monoclonal antibody (mAb, MultiSciences, Hangzhou, China).

### SPR-Based Binding Assay

A surface plasmon resonance (SPR) assay was conducted to assess the binding ability of Z_HPV16E6_ affibodies to recombinant HPV16 E6 on a BIAcore T200 (GE Healthcare, Uppsala, Sweden) as previously described (Zhu J. et al., 2020). As a negative control, affibody Z_WT_ was used. With a 1:1 L binding model, data on SPR were fit globally and evaluated via BIA 3.0.2 software.

### Immunofluorescence Staining

An indirect immunofluorescence assay (IFA) was performed following previously protocols (Zhu J. et al., 2020). Briefly, we evenly cultured TC-1, CaSki, HeLa229, and C666-1 cells on coverslips in 6-well cell culture plates, and subjected them to a 6-h incubation with the Z_HPV16E6_ affibodies or Z_WT_ affibody control, with a final concentration at 100 μg/ml. After five times PBS wash aimed to eliminate the free molecules, we then fixed the cells with 4% paraformaldehyde for 20 min at room temperature and permeabilized to promote the binding of the primary (mouse anti-His-tag mAb) and secondary [fluorescin thiocyanate (FITC)-conjugated goat anti-mouse IgG (H + L)] antibodies (Life Technologies, Carlsbad, CA, United States). Exactly 50 μM propidium iodide (PI; Beyotime Biotech Co., Ltd., China) was used for cell nuclei staining at 37°C for 10 min, and images were obtained with a confocal fluorescence microscope (Nikon C1-i, Japan).

### Cervical Cancer Tissue Sample Collection

Eight cervical cancer tissue specimens were obtained from HPV 16-positive cervical cancer patients in the Department of Pathology, The First Affiliation Hospital of Wenzhou Medical University. In addition, 5 samples were collected from normal cervixes, proven negative for HPV by PCR, and used as negative controls. The project was approved by the Scientific and Ethical Committee of The First Affiliation Hospital of Wenzhou Medical University.

### Immunohistochemical Staining

Three-micrometer sections were cut from the tissue blocks, placed on glass slides, baked at 60°C for 60 min, deparaffinized, and rehydrated through graded alcohol rinses. Then, the slides were immersed in Tris/EDTA pH 9.0 buffer (10 mM Tris, 1 mM EDTA) and subjected to high-pressure processing for 3 min for heat-induced antigen retrieval. After washing in tap water, non-specific site blocking was followed by the addition of 10% normal goat serum in PBS buffer. Then, the slides were treated with 0.3% H_2_O_2_ in methanol for 10 min to inactivate endogenous peroxidase activity. Subsequently, the tissues were incubated with Z_HPV16E6_ affibodies (100 μg/ml) for 1.5 h at 37°C and then with mouse anti-His-tag mAb overnight at 4°C and HRP-conjugated goat-anti-mouse antibody at 37°C for 1.5 h. In addition, rabbit anti-HPV16 E6 polyclonal (prepared in-house) was used as a positive control, and Z_WT_ and PBS were used as negative controls.

### Labeling of Affibody Molecules With DyLight 755

The labeling of Z_HPV16E6_ affibodies with DyLight 755 (Thermo Fisher Scientic, United States) was carried out in accordance with the manufacturer’s instructions. The labeled affibody molecules were confirmed by SDS-PAGE and further detected at wavelengths of 730–950 nm by an *in vivo* fluorescence imaging system (CRi Maestro 2.10, United States; Supplementary Figure 3A).

### Biodistribution in Tumor-Bearing Mice

The tumor targeting ability and dynamic distribution of the Z_HPV16E6_ affibodies were investigated in nude mice using NIR optical imaging. In brief, 1 × 10^6^ TC-1 and HeLa229 cells were injected subcutaneously into the upper axillary fossa of nude mice (at least three mice per group). When the volume reached 300 ∼ 500 mm^3^, mice received an intravenous injection of DyLight 755-conjugated Z_HPV16E6_ affibodies (100 μg; 150 μL per mouse). Imaging was carried out at different time points post-injection (pi) using the NIR Imaging System (Cri Maestro 2.10, United States). To confirm whether uptake was mediated by specific targeting of HPV16 E6, HPV16-negative xenografts treated with Z_HPV16E6_ affibodies and HPV16-positive xenografts treated with Z_WT_ affibody were used as negative controls. In addition, the tumor/skin ratios = (tumor signal – background signal)/(skin signal – background signal) × 100% at different pi time points were analyzed.

### Western Blotting Analysis

CaSki cells seeded at 1 × 10^5^ per well in 6-well plates were incubated with medium containing either Z_HPV16E6_1235 or Z_WT_ or medium alone for the indicated time. Equal amounts of protein (30 μg) were evaluated using the Western blot technique with reference to a previously highlighted protocol (Zhu J. et al., 2020). In addition, we have listed all the primary antibodies in Supplementary Table 1.

### Immunoprecipitation

CaSki cells (6 × 10^5^) were plated in 10-cm tissue culture dishes and treated with 10 μM Z_HPV16E6_1235 or Z_WT_ control for 24 h. Following washes with PBS, pellets were lysed on ice for 20 min with cell lysis buffer (Beyotime Biotechnology, Shanghai, China) supplemented with protease inhibitors (Roche Molecular Biochemicals, Indianapolis, IN, United States) and cleared by centrifugation at 12,000 × *g* for 20 min at 4°C. Then, 5 μg anti-p53 antibody was added to 400 μg whole cell lysates and gently rotated at 4°C overnight. According to the manufacturer’s instructions, the immunocomplex was collected with Protein A/G agarose (Beyotime Biotechnology, Shanghai, China). Finally, proteins were released by boiling in reducing SDS sample buffer and analyzed by Western blotting.

### Efficacy of the Combination of Z_HPV16E6_1235 and Z_HPV16E7_384 *in vitro*

The Cell Counting Kit-8 (CCK-8) assay was performed to evaluate the efficacy of Z_HPV16E6_1235 and Z_HPV16E7_384, alone and in combination, in TC-1 and Caski (HPV16 positive) cells. Briefly, 5 × 10^3^ cells were seeded onto 96-well plates and subsequently incubated for 2 days with the indicated agents at increasing concentrations (0.5, 1, 2.5, 5, 10, and 20 μM). HPV16-positive cells treated with the Z_WT_ affibody and HPV16-negative cells (including HPV18-positive HeLa229 cells and HPV-negative C666-1 cells) treated with the indicated agents were used as negative controls. Next, CCK-8 solution (10 μL, Dojindo, Japan) was put into every well and subjected to another 30 min-incubation. Using a microplate reader, we measured absorbance at 450 nm, from which cell viability was determined. The half maximal inhibitory concentration (IC50) values were calculated using GraphPad Prism software (GraphPad Software, Inc.). The CCK−8 assay was performed at least three times.

### Plate Colony Formation Assay

Experiments for plate colony formation were also conducted for cell proliferation ability analysis. Briefly, we seeded cells (5 × 10^3^) in a 6-well culture plate. Cultures were maintained in a medium comprised of the indicated agents or medium alone for 14 days. Then, after cell fixation, a 20 min-staining was conducted with 0.1% crystal violet (Amresco, Solon, OH, United States). Images of the stained colonies were taken and counted.

### Chou-Talalay Analysis

Pharmacological interaction between Z_HPV16E6_1235 and Z_HPV16E7_384 was determined using Chou-Talalay analysis (Chou, 2010). Briefly, 5 × 10^3^ cells were seeded onto 96-well plates and subsequently incubated for 2 days with the indicated agents at different concentration combinations. Then, a CCK8 assay was used to measure the combined effect of the two therapeutic agents from different groups on TC-1 and CaSki cells. Using Compusyn software^2^, dose-effect curves for each regimen and for the combination of agents were plotted and an estimate of the combination index (CI) is achieved. A CI of >1, <1, = 1 denotes antagonism, synergy, and additive effects, respectively.

### Flow Cytometric Analysis and Senescence-Associated ß-Galactosidase Assay

Cell cycle alteration and apoptosis induction were evaluated using Flow cytometry analysis in HPV16-positive cell lines following exposure to Z_HPV16E6_1235 and Z_HPV16E7_384 alone and in combination. PI (MultiSciences, Hangzhou, China) was used to stain the cells for cell cycle analysis as described by the manufacturer’s instructions and analyzed by flow cytometry (BD Biosciences, SanJose, CA, United States). G0/G1, S, and G2/M phase percentages were calculated and compared using ModFit LT 3.0 software. PI and Annexin V-FITC (Invitrogen, Carlsbad, CA, United States) were used to stain the apoptotic cells as described by the manufacturer’s recommendations and quantified using flow cytometry (CytoFLEX, Beckman Coulter, United States). Cells were categorized into viable, dead, early apoptotic, and apoptotic cells and the ratio of apoptotic (including early apoptotic) cells were compared with the control for each experiment.

For senescence analysis, the cells were fixed in 2% formaldehyde/0.2% glutaraldehyde and stained using X-Gal (5-bromo-4-chloro-3-indolyl-galactopyranoside, Beyotime Biotechnology, Shanghai, China) at pH of 6.0 as described by manufacturer’s instructions. SA-β-Gal-positive cells were counted in three representative fields.

### Statistical Analysis

Data were presented as mean ± standard deviation (SD). Statistical analysis of the significance between groups was conducted using 2-tailed unpaired Student’s test, and *P* < 0.05 was considered to be statistically significant. All calculations were performed with the software SPSS16.0.

## Results

### Generation and Purification of Z_HPV16E6_ Affibodies

DNA sequencing was performed on 66 ELISA-positive clones (OD > 0.5, Supplementary Figure 1) after three rounds of screening of a combinatorial affibody library and identified 42 unique phagemid inserts, occurring one to seven times. As shown in Supplementary Figure 2, these 42 clones showed high homology in the framework region of the original affibody scaffold molecule Z_WT_ but were highly diverse in the 1 and 2 helical regions. Three potential affibodies, Z_HPV16E6_1115, Z_HPV16E6_1171, and Z_HPV16E6_1235, were selected for further analysis on the basis of the following criteria: (a) high binding affinity in the ELISA screening, (b) appearance frequency for the particular clone, and (c) relatively high-yield expression and purification as recombinant proteins in *E. coli BL21(DE3)*.

**FIGURE 1:**
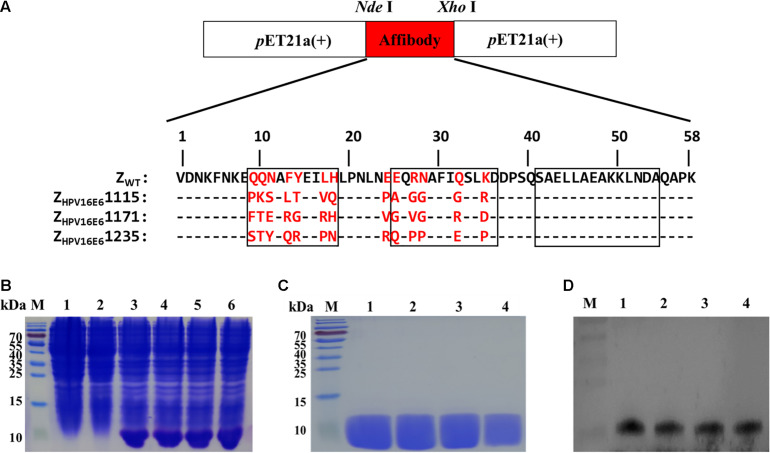
Production of Z_HPV16E6_ affibodies. **(A)** The recombinant plasmids of *p*ET21a(+)/Z_HPV16E6_ affibodies and *p*ET21a(+)/Zwt affibody were constructed using the *Nde*I and *Xho*I restriction sites and further confirmed by DNA sequencing. Amino acid sequences of the Z_WT_ aligned to 3 selected Z_HPV16E6_ affibodies. The 13 randomized amino acid positions are highlighted in red. Dashes (–) indicate amino acid identities, and black boxes show three α-helical subdomains in the wild-type Z domain. **(B)** Coomassie Brilliant Blue-stained SDS-PAGE gel of the recombinant proteins in *E. coli* BL21 induced by 1 mM IPTG. M, protein ladder; 1, empty *E. coli* BL21(DE3); 2, *E. coli* BL21(DE3) transformed with *p*ET21a(+) empty vector; 3–6, *E. coli* BL21(DE3) transformed with *p*ET21a(+)/Z_HPV16E6_1115, *p*ET21a(+)/Z_HPV16E6_1171, *p*ET21a(+)/Z_HPV16E6_ 1235, and *p*ET21a(+)/Z_WT_ plasmid, respectively. The purified Z_HPV16E6_ affibodies were evaluated by 15% SDS-PAGE **(C)** and verified by Western blotting **(D)** M, protein marker; 1, Z_HPV16E6_1115; 2, Z_HPV16E6_1171; 3, Z_HPV16E6_1235; and 4, Z_WT_.

The three affibody genes were then inserted into *p*ET21a (+) using the *Nde*I and *Xho*I restriction sites to generate the recombinant plasmid *p*ET21a(+)/Z_HPV16E6_ (Figure 1A). The resulting plasmids were transformed into *E. coli BL21(DE3)* and further induced to express recombinant His-tag fusion affibody protein by the addition of 1 mM IPTG for 6 h at 37°C. As shown in Figure 1B, channels 3–6, the band was detected at 6.5 kDa in *E. coli BL21(DE3)* transformed with *p*ET21a(+)/affibody after induction, which was consistent with the expected size of the affibody molecules. However, in the test, *E. coli BL21(DE3)* bacteria alone and *E. coli BL21(DE3)* transformed with *p*ET21a(+) empty vector did not show the 6.5 kDa band, indicating that the bacteria itself and the *p*ET21a(+) empty vector did not express the protein (Figure 1B, channel 1–2). After the successful induction of *p*ET21a(+)/Z_HPV16E6_ expression, we further purified His-tag fusion affibodies by affinity chromatography using Ni-NTA agarose resin. SDS-PAGE analysis showed a distinct band of the expected molecular mass, indicating that the final affibody molecules were pure and stable (Figure 1C). In addition, Western blotting results showed that these purified proteins could specifically react with mouse anti-His-tag mAb (Figure 1D).

### Biosensor Binding Analyses of Z_HPV16E6_ Affibodies

**FIGURE 2:**
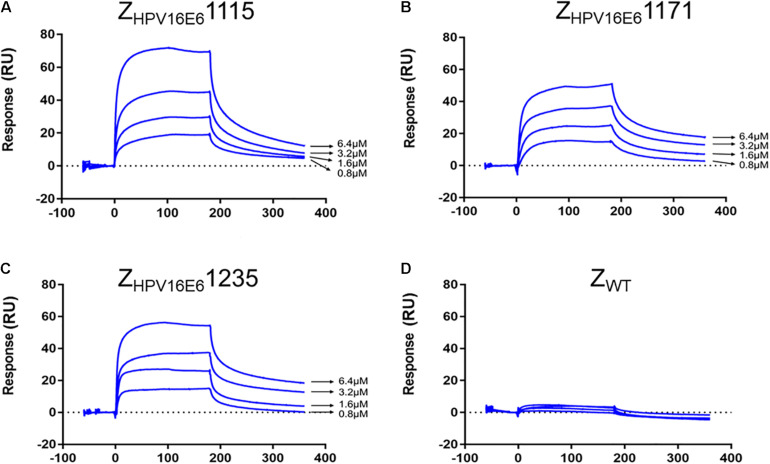
SPR analysis of the interaction between Z_HPV16E6_ affibodies and the immobilized HPV16 E6 target ligand. Sensorgram obtained after injection of different concentrations of purified Z_HPV16E6_1115 **(A)** Z_HPV16E6_1171 **(B)** and Z_HPV16E6_1235 **(C)** over the surface with immobilized recombinant HPV16 E6. Z_WT_ affibody was set as a control **(D)**.

The real-time biospecific interaction of the selected affibody molecule with the target protein was investigated using a BIAcore T200 instrument. The target protein, HPV16 E6, was immobilized on the carboxylate glucan surfaces in a CM5 chip, and different amounts of Z_HPV16E6_ (i.e., 0.8 to 6.4 μM) passed through the chip at 30 μL/min at 25°C. As shown in Figures 2A–C, concentration-dependent increases in resonance signals were detected, suggesting that the three affibodies bound well to recombinant HPV16 E6. As expected, Z_WT_ control could not be detected in any effective reaction units in resonance signals (Figure 2D). To further determine the dissociation equilibrium constant (KD), the association rate constant (k_on_), and the dissociation rate constant (k_off_), kinetic BIAcore analysis was performed using BIA evaluation 3.0.2 software (Biacore) through a one-to-one Langmuir binding model. Analysis results showed that the dissociation equilibrium constant (KD) values of Z_HPV16E6_1115, Z_HPV16E6_1171, and Z_HPV16E6_1235 were 5.475E–06 mol/L, 6.229E–05 mol/L, and 1.280E–07 mol/L, respectively, which were significantly lower than that of the Z_WT_ affibody (1.404E–00 mol/L). Conversely, the association rate constants (K_on_) values of the three affibody molecules were significantly higher than that of the Z_WT_ affibody (Table 1). SPR data demonstrated very clearly that all three Z_HPV16E6_ affibodies we selected had high binding affinity to recombinant HPV16 E6.

**TABLE 1:** Kinetic data from the SPR biosensor analysis of the Affibody Molecules.

	Kon(1/Ms)	Koff(1/s)	KD(M)
Z_HPV16E6_1115	3.50E + 02	1.92E–03	5.475E–06
Z_HPV16E6_1171	1.29E + 02	8.06E–03	6.229E–05
Z_HPV16E6_1235	1.68E + 02	2.15E–05	1.280E–07
Z_WT_	6.31E–04	8.86E–04	1.404E–00

### Specificity Analysis of Z_HPV16E6_ Affibodies

**FIGURE 3:**
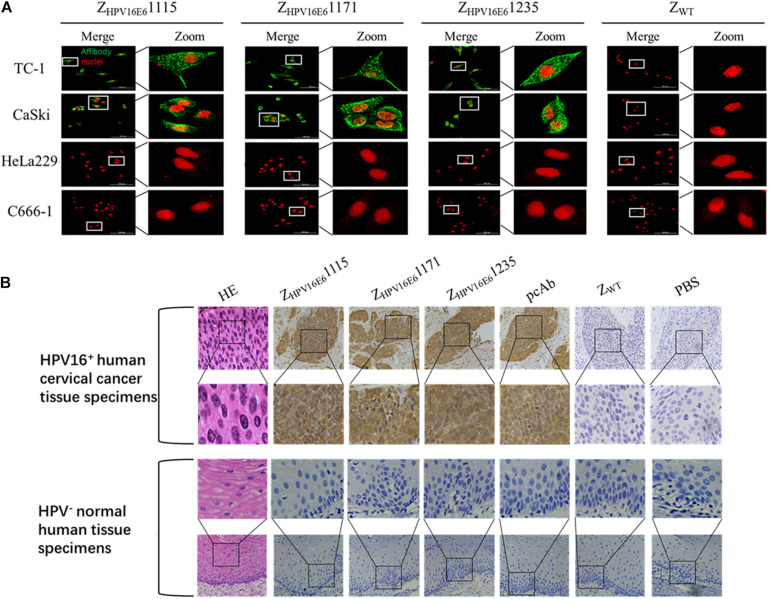
Immunofluorescence and immunohistochemical staining analyses of the binding specificity of Z_HPV16E6_ affibodies to HPV16 E6. **(A)** Representative images showing TC-1, CaSki cells (HPV16 positive), HeLa229 (HPV18 positive), and C666-1 cells (HPV negative) stained with Z_HPV16E6_1115, Z_HPV16E6_1171, and Z_HPV16E6_1235. The Z_WT_ affibody was used as a negative control. The affibody molecule stain is shown in green, while the nuclear stain (PI) is shown in red; magnification at ×400. **(B)** Representative image of HPV16-positive cervical cancer sections and HPV-negative normal human sections by hematoxylin and eosin (HE) staining and immunohistochemistry (IHC) staining with Z_HPV16E6_ affibodies. Sections from HPV16-positive cervical cancer sections (upper panel) and HPV-negative normal human sections (lower panel) were labeled with Z_HPV16E6_ affibodies. Polyclonal HPV16 E6 antibody was used as a positive control. Z_WT_ and PBS were used as negative controls. Magnification at ×400.

After confirming the binding ability of Z_HPV16E6_ to recombinant HPV16 E6, we next investigated whether Z_HPV16E6_ affibodies could specifically bind to native HPV16 E6 expressed in HPV16-positive cells. We therefore incubated Z_HPV16E6_ affibodies or Z_WT_ control with live cells for 6 h and analyzed by IFA using confocal microscopy (400 × magnification). The results showed a large number of green dots in a punctate pattern inside HPV16-positive TC-1 and CaSki cells, suggesting efficient internalization of the 6.5-kDa affibody molecule and targeting of HPV16-positive cells. In contrast, HPV16-negative cells (including HPV18-positive HeLa229 cells and HPV-negative C666-1 cells) treated with Z_HPV16E6_ affibodies and HPV16-positive cells treated with Z_WT_ did not produce any fluorescent signal after the same duration of incubation (Figure 3A). Moreover, the immunohistochemistry (IHC) assay offered additional evidence for a specific interaction of the Z_HPV16E6_ affibodies with HPV16 E6. All three Z_HPV16E6_ affibodies functioned very well as detection reagents and showed brown signals in HPV16-positive human cervical cancer tissue specimens but not in HPV-negative normal human tissue specimens, which concur to the staining pattern of the anti-HPV16 E6 polyclonal antibody (Figure 3B). These results revealed that the Z_HPV16E6_ affibodies exhibit strong specific binding to native HPV16 E6 expressed in HPV16-positive cell lines and tissues.

### Tumor Targeting Ability of Z_HPV16E6_ Affibodies *in vivo*

**FIGURE 4:**
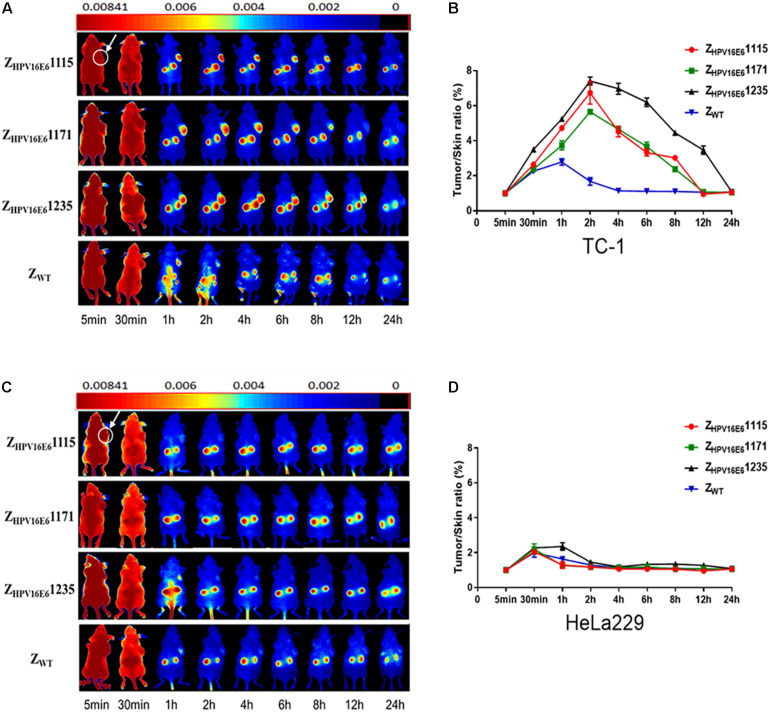
*In vivo* testing of the target binding ability of Z_HPV16E6_ affibodies to tumor tissues. Tumor imaging in nude mice bearing TC-1 **(A)** or HeLa229 **(C)** xenografts (arrows) by using fluorescence-conjugated affibody molecules. NIR-based imaging was executed at various time points pi with DyLight 755-conjugated Z_HPV16E6_ affibodies and used DyLight 755-conjugated Z_WT_ affibody as a negative control. Tumor-to-skin ratios were calculated at different time points pi of the indicated agents in nude mice bearing TC-1 **(B)** and HeLa229 **(D)** xenografts. Data are shown as the mean ± SD of triplicates.

Encouraged by the impressive results obtained *in vitro*, we further investigated whether the Z_HPV16E6_ affibodies could also efficaciously and specifically accumulate in HPV16-positive tumor xenografts *in vivo* by using DyLight 755-labeled affibody molecules. Athymic nude mice carrying TC-1 (HPV16 positive) or HeLa229 (HPV18 positive) xenografts were injected with DyLight 755-conjugated Z_HPV16E6_ affibodies or Z_WT_. A near-infrared fluorescence (NIR) optical imaging system was used to determine the *in vivo* biodistribution and tumor uptake efficacy of the Z_HPV16E6_ affibodies over a time course of 5 min to 72 h. As shown in Figure 4A, the fluorescence signal of DyLight 755-Z_HPV16E6_ affibodies in the TC-1 xenograft model was detectable as early as 30 min post injection. Subsequently, high-contrast fluorescent signals were obtained 1 h post injection (hpi), peaked at 2 hpi, and remained steady for over 8 hpi with DyLight 755-Z_HPV16E6_1115 and DyLight 755-Z_HPV16E6_1171 and over 12 hpi with DyLight 755-Z_HPV16E6_1235 (Figures 4A,B). However, in the HeLa229 xenograft model, a non-specific fluorescent signal of DyLight 755-labeled Z_HPV16E6_ affibodies in the tumor was observed at 30 min pi and cleared within 1–2 h, which is similar to the results in the xenograft model (both TC-1 and HeLa229 cells) treated with DyLight 755-Z_WT_ control (Figure 4A, Z_WT_ panel and Figure 4C). In addition, affibody molecular accumulation in the kidneys was observed in athymic nude mice with or without tumor xenografts, indicating that the small DyLight 755-labeled affibody proteins were cleared by kidney filtration (Figure 4 and Supplementary Figures 3B,C). Since Z_HPV16E6_1235 has a better affinity for SPR detection and residence time in the mouse body than the other two Z_HPV16E6_ affibodies, Z_HPV16E6_1235 was selected for further research.

### Affibody Z_HPV16E6_1235 Restores the Intracellular Expression and Transcriptional Activity of p53 in HPV16-Positive Cells

**FIGURE 5:**
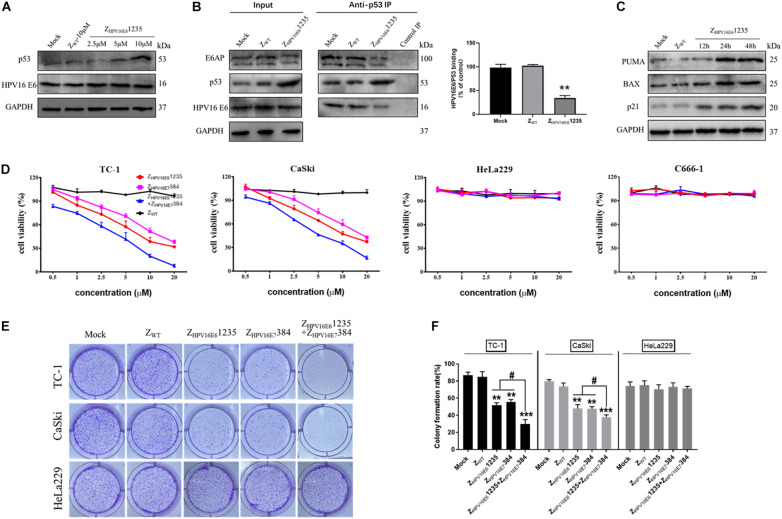
Z_HPV16E6_1235 inhibits the proliferation of HPV16-positive cell lines by binding to and blocking the intracellular activity of the HPV16 E6 oncoprotein. **(A)** p53 was up-regulated in a dose-dependent manner by treatment with Z_HPV16E6_1235 in CaSki cells. **(B)** The effect of Z_HPV16E6_1235 on the intracellular binding between HPV16 E6 and p53 was assessed by immunoprecipitating the E6/E6AP/p53 trimeric complex using an anti-p53 antibody bound to Protein A/G agarose beads from CaSki cells treated for 24 h. A parallel negative control assay was run for each group by incubating cell lysates with control IgG. The bar graph represents the amount of E6 bound to the relative amount of immunoprecipitated p53 after the quantification of p53 and E6 protein bands with ImageJ software. Data are presented as the mean ± SD of three independent experiments. **P < 0.01. **(C)** CaSki cells were treated with 10 μM Z_HPV16E6_1235 for the indicated periods and the expression of p53 target genes, including PUMA, BAX and p21, was evaluated by Western blotting. Cells without any treatment (Mock) or treated with 10 μM Z_WT_ for 48 h were used as negative controls. GAPDH served as an internal reference standard. **(D)** The effects of Z_HPV16E6_1235 and Z_HPV16E7_384 alone or in combination on the viability of HPV16-positive cancer cells (TC-1, CaSki), HPV18-positive cervical cancer cells (HeLa229) and HPV-negative cancer cells (C666-1) were assessed by CCK-8 assay after 48 h of treatment with the indicated concentrations; these cells were compared to Z_WT_-treated cells. Data are shown as the mean ± SD of three independent experiments. **(E–F)** Colony formation assays of HPV16-positive TC-1 and CaSki cells or HPV18-positive HeLa229 cells following treatment with 2.5 μM test affibody molecules for 14 days. The Z_WT_ affibody and medium groups were set as controls. **P < 0.01, ***P < 0.001 vs. the control group. ^#^*P* < 0.05 vs. the Z_HPV16E6_1235 or Z_HPV16E7_384 alone treatment group.

Given that the affibody molecule investigated was able to bind HPV16 E6 with high binding affinity and specificity, we next asked whether targeting HPV16 E6 with Z_HPV16E6_1235 could protect p53 from HPV16 E6-mediated degradation in cells endogenously expressing HPV16 E6 (i.e., CaSki, HPV16-positive). As reported in Figure 5A, the Western blotting results showed that incubation with Z_HPV16E6_1235 for 24 h was indeed capable of impeding the degradation of p53 induced by HPV16 E6, as indicated by a marked increase in p53 expression level in HPV16-positive CaSki cells in a concentration-dependent manner. In contrast, treatment with Z_WT_ failed to result in the accumulation of p53, further underlining the specific activity of Z_HPV16E6_1235 in HPV16-positive cells. To further validate that Z_HPV16E6_1235 exerts biological activity in HPV16-positive cells through impeding the physical interaction between HPV16 E6 and p53, we performed coimmunoprecipitation (IP) experiments and analyzed the relative amount of HPV16 E6 and E6AP bound to p53 by Western blotting. For this purpose, CaSki cells were treated with 10 μM Z_HPV16E6_1235 or Zwt control for 24 h, followed by IP with an anti-p53 antibody. As shown in Figure 5B, Z_HPV16E6_1235 significantly decreased the amount of HPV16 E6 and E6AP that coimmunoprecipitated with p53 in Z_HPV16E6_1235-treated cells compared to the control-treated cells (mock or Z_WT_), suggesting that Z_HPV16E6_1235 can directly block HPV16 E6/p53 binding.

We then focused on whether Z_HPV16E6_1235 might be able to restore the transcriptional activity of p53. Toward this aim, Western blotting assays were performed to detect the expression of some known p53 target genes, including BAX, BBC3 (PUMA), and CDKN1A (p21), which are closely related to apoptosis and cell cycle arrest. The results showed that compared to treatment with the control (mock or Z_WT_), Z_HPV16E6_1235 significantly up-regulated the expression of PUMA, BAX, and p21 in a time-dependent manner, suggesting that the restored p53 protein is functionally active (Figure 5C). Taken together, these results demonstrate that affibody Z_HPV16E6_1235 can rescue both the expression and transcriptional activity of p53 in HPV16-positive cancer cells.

### Synergistic Inhibition of HPV16-Positive Cell Growth by Combination Treatment With Z_HPV16E6_1235 and Z_HPV16E7_384

The high level of both p53 and the activation of its target genes (BAX, PUMA, and p21) implied a pro-death program in HPV16-positive cells treated with Z_HPV16E6_1235, which prompted us to verify whether Z_HPV16E6_1235 affects the proliferation of HPV16-positive cells. Additionally, we previously reported that targeting HPV16 E7 with affibody Z_HPV16E7_384 had significant *in vivo* antitumor efficacy (Jiang et al., 2018). Therefore, we were interested in whether targeting E6 or simultaneously targeting E6 and E7 could inhibit the proliferation of HPV16-positive cells more effectively than targeting the E7 oncoprotein alone. CCK-8 assays showed that the cell viability of two HPV16-positive tumoral cell lines was inhibited by Z_HPV16E6_1235 over the range of 0.5 to 20 μM compared to that in control cells, which was similar to that with Z_HPV16E7_384 treatment (Figure 5D). Of note, we also detected that the combination of Z_HPV16E6_1235 and Z_HPV16E7_384 therapy was significantly superior to either agent used alone at the same concentration in HPV16-positive cell lines (Figure 5D), and these results were further confirmed by long-term colony formation assays (Figures 5E,F), which are regarded as the “gold standard” for measuring cellular sensitivity to drug treatment. As expected, HPV16-positive cells treated with the Z_WT_ affibody and HPV18-positive cells treated with Z_HPV16E6_1235 and Z_HPV16E7_384 alone or in combination remained fully viable (Figures 5D–F). Following statistical analysis, the half-maximal inhibitory concentration (IC50) values for Z_HPV16E6_1235 alone, Z_HPV16E7_384 alone, or the combination in TC-1 cells were 7.202, 11.460, and 3.071 μM, respectively. In Caski cells, these values were 9.975, 14.480, and 4.843 μM.

To further determine if Z_HPV16E6_1235 and Z_HPV16E7_384 combination therapy had synergistic, additive, or antagonistic effects in TC-1 and CaSki cells, Chou-Talalay methods was used. Based on the IC50 values, the synergistic activity of Z_HPV16E6_1235 (1, 5, or 10 μM) and Z_HPV16E7_384 (10 μM) was proved to be statistically significant (Table 2). These findings thus validate that Z_HPV16E6_1235 synergizes with Z_HPV16E7_384 to induce a greater therapeutic effect on HPV16-positive cell lines than either therapy alone.

**TABLE 2:** The synergistic therapeutic effect of ZHPV16E61235 in combination with ZHPV16E7384 was demonstrated by Chou-Talalay analysis.

Z_HPV16E6_1235 (μM)	Z_HPV16E7_384 (μM)	Cytotoxicity (%)	Cl
lnTC-1 cell line			
1	10	58	0.738
5	10	73	0.563
10	10	84	0.389
In CaSki cell line			
1	10	55	0.810
5	10	67	0.812
10	10	80	0.656

### Effects of Z_HPV16E6_1235 in Combination With Z_HPV16E7_384 on the Cell Cycle, Apoptosis, and Cellular Senescence

**FIGURE 6:**
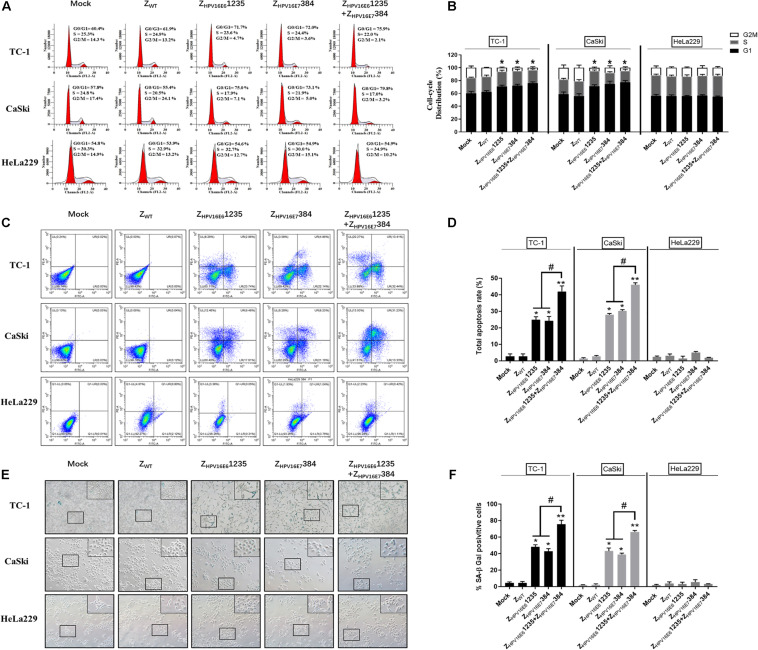
Effect of Z_HPV16E6_1235 in combination with Z_HPV16E7_384 on the cell cycle, apoptosis, and cellular senescence. TC-1 and CaSki cells were treated with 10 μM of test affibody molecules for 24 h and analyzed by flow cytometry assay for the cell cycle **(A,B)** and apoptosis **(C,D)** with PI and Annexin V/PI. Data are presented as the mean ± SD in three independent experiments. **(E,F)** SA-β-gal staining of TC-1 and CaSki cells treated with 10 μM test affibody molecules for 24 h. Representative images taken using a bright-field inverted microscope (100 × magnification) are shown. In all panels, TC-1 and CaSki (HPV16-positive) cells treated with Z_WT_ and HeLa229 (HPV18-positive) treated with selected affibodies were used as negative controls. Significance: *P < 0.05, **P < 0.01 vs. the control group. ^#^*P* < 0.05 vs. the Z_HPV16E6_1235 or Z_HPV16E7_384 alone treatment group.

To uncover the potential mechanism of the inhibitory effect of targeting HPV16 E6 and/or E7 on HPV16-positive cancer cell proliferation, flow cytometric analysis and senescence-associated β-galactosidase (SA-β-Gal) were performed. As shown in Figures 6A,B, HPV16-positive (TC-1 and CaSki) cells treated with Z_HPV16E6_1235 and Z_HPV16E7_384 alone or in combination all showed significant cell cycle arrest at the G0-G1 phase compared to cells treated with Zwt. However, there was no obvious difference between the combination treatment group and either single-agent group in terms of the number of G0/G1 phase cells, indicating that the synergistic antiproliferative effect may not be related to cell cycle arrest. Subsequent experiments were performed to evaluate cell apoptosis *in vitro*. Figures 6C,D shows that treatment with Z_HPV16E6_1235 resulted in an approximately 25% increase in apoptotic HPV16-positive cells, which was similar to the treatment with Z_HPV16E7_384. Notably, Z_HPV16E6_1235 in combination with Z_HPV16E7_384 significantly elevated cell apoptosis to levels greater than those observed with single-agent treatments (Figures 6C,D).

Next, we examined whether the combination of Z_HPV16E6_1235 and Z_HPV16E7_384 could more effectively induce senescence in TC-1 and CaSki cells. SA-β-Gal activity was assessed 24 h after exposure to the combination of Z_HPV16E6_1235 and Z_HPV16E7_384. Significantly higher SA-β-Gal activity was observed after treatment with the combination compared to either agent alone, as indicated by strong blue staining. However, exposure to Z_HPV16E6_1235 and Z_HPV16E7_384 alone resulted in moderate SA-β-Gal activity. The ratio of SA-β-Gal-positive cells is summarized and shown in Figures 6E,F. Taken together, these data revealed that the synergistic antitumor activity may be mainly attributed to the induction of cellular senescence and apoptosis but is not related to cell cycle arrest.

## Discussion

Although HPV-related cancers can be prevented to a great extent by prophylactic HPV vaccines that are commercially available, these vaccines have little preventive or therapeutic effects against pre-existing HPV infections. Additionally, a considerably long time would be needed for preventive vaccines to lower the incidence of cervical cancer owing to the limited use of prophylactic HPV vaccines attributed to high costs and medical infrastructure challenges (Herrero et al., 2015). Therefore, the research and development of effective diagnostic and therapeutic strategies for HPV-related cancer, such as molecular imaging and targeted tumor therapy, are urgently needed.

Affinity proteins are invaluable tools in the advancement of next-generation imaging and therapeutic agents (Löfblom et al., 2010). To date, monoclonal antibodies (mAbs) are the most widespread and successful affinity proteins for life science applications. However, due to their large mass (∼150 kDa), mAbs are associated with several intrinsic drawbacks, including poor tissue-penetrating ability and a long residence circulation time, which lead to poor imaging quality (Tolmachev et al., 2010). In comparison to mAbs, affibody molecules, a novel category of affinity proteins, are very small in molecular size (∼6.5 kDa) and hence have favorable properties for imaging diagnostic and various biological applications (Löfblom et al., 2010; Ståhl et al., 2017; Gebauer and Skerra, 2020; Tolmachev and Orlova, 2020). Recently, human clinical trials strikingly confirmed that HER2-specific affibody molecules labeled with ^111^In can be used for targeted detection of HER2 overexpression in metastatic breast cancer using single-photon emission computed tomography (SPECT; Baum et al., 2010). Because of their simple structure and small size, affibody molecules are readily produced by conventional peptide synthesis or bacterial fermentation methods, which could greatly facilitate the manufacturing process and clinical application. In the present study, we conducted biopanning, phage-ELISA screening and DNA sequencing from a combinatorial phage library to obtain three potential HPV16 E6-binding affibody molecules (Z_HPV16E6_1115, Z_HPV16E6_1171, and Z_HPV16E6_1235). We then successfully produced these affibody molecules with high purity and solubility in a prokaryotic expression system. High target-binding affinity is an important feature for the successful application of a novel affinity protein candidate in tumor diagnosis and therapy. Our work showed that by SPR, the binding affinity of all three Z_HPV16E6_ affibodies selected in this study to HPV16 E6 was approximately 10^6^ times higher than that of Zwt. Moreover, indirect IFAs showed bright spotty or patchy fluorescence in HPV16-positive cell lines only, and the specificity was further supported by immunohistochemical staining analysis. Of note, in tumor-bearing nude mice, Z_HPV16E6_ affibodies were capable of target-specific accumulation in HPV16-positive xenografts, highlighting that the Z_HPV16E6_ affibodies may be promising candidates for molecular imaging.

In tumor imaging and diagnosis, the rapid internalization of imaging traces by cancer cells and the efficient clearance of unbound tracking agents by excretory organs are other important properties for ideal probes to provide high-contrast tumor imaging. Dynamic optical imaging results showed that DyLight 755-labeled Z_HPV16E6_ affibodies circulated to tumor tissues as early as 30 min pi. These affibodies quickly and specifically accumulated for clear, high-contrast tumor imaging within 2 h and were retained in tumors for over 8–12 h. In addition, similar to the observations of several previous studies (Orlova et al., 2006; Xue et al., 2016; Jiang et al., 2018; Zhu J. et al., 2020; Zhu S. et al., 2020), Z_HPV16E6_ affibodies were also detectable in the kidneys. This could be explained by the passage of small proteins through the glomerular membrane, which is eventually absorbed by the proximal tubules (Behr et al., 1998; Vegt et al., 2010; Wang et al., 2019). Also, the high affibody levels in the kidney could be attributed to the phenomenon that proteins less than 60 kDa are typically cleared by the renal (Wang et al., 2019). Taken together, these characteristics strongly imply the favorability of Z_HPV16E6_ affibodies for molecular imaging and may improve the early diagnosis of HPV-related cancer for appropriate treatment choices.

Thus far, we have demonstrated that Z_HPV16E6_ affibodies can specifically target the HPV16 E6 oncoprotein with high affinity; in addition, it is necessary to discuss whether affibody molecules targeting HPV16 E6 can block its intracellular activity. In epithelial tumors induced by HR-HPV, including cervical carcinoma and head and neck tumors, p53 is degraded by the E6 viral oncoprotein (Scheffner et al., 1990). In this process, E6 binds to a short leucine (*L*)-rich LxxLL consensus sequence within the cellular ubiquitin ligase E6AP3. Subsequently, the E6/E6AP heterodimer recruits p53 for proteasome-mediated degradation, ultimately leading to cell immortalization and cancer development (Scheffner et al., 1990; Martinez-Zapien et al., 2016; Li et al., 2019). Therefore, interrupting E6/E6AP/p53 trimeric complex formation and impeding p53 degradation by E6 offer an interesting therapeutic option for HPV-related tumors. A recent study reported that one linear short peptide, which could selectively bind to the HPV16 E6 oncoprotein, restored the expression of functional p53 protein and specifically killed HPV16-positive cervical cancer cells by inducing apoptotic cell death (Celegato et al., 2020). Similar to previous reports (Dymalla et al., 2009; Celegato et al., 2020), treatment with Z_HPV__16E6_1235 significantly elevated the expression of p53 in HPV16-positive cancer cells compared with treatment with Z_WT_. Subsequent studies verified that the restored p53 induced by Z_HPV16E6_1235 treatment is transcriptionally active, leading to an obvious upregulation of p53 target genes, in particular the proapoptotic genes BAX and PUMA and genes related to cell cycle arrest and senescence, such as p21. Given the accumulation of p53 in cancer cells, we further explored the influence of Z_HPV16E6_1235 on the phenotype of HPV16-positive cell lines. Consistent with the accumulation of p53 in cancer cells, treatment with Z_HPV16E6_1235 specifically inhibited cell viability and proliferation without causing cytotoxicity in other unrelated cells. Moreover, cell-cycle distribution analysis showed that treatment with Z_HPV16E6_1235 led to an increased accumulation of G0/G1 cells, together with a remarkable decrease in G_2_/M cells, compared with the control (Mock and Z_WT_). Subsequent studies also showed that more apoptotic and senescent tumor cells were observed in the Z_HPV16E6_1235 treatment group than the control group. Therefore, we suggested that the reduction in cell proliferation induced by treatment with Z_HPV16E6_1235 is potentially associated with cell cycle G0/G1 phase arrest, apoptosis and senescence in HPV16-positive cell lines.

It is well accepted that the functional inactivation of p53 and Rb tumor suppressor proteins by the HPV E6 and E7 oncoproteins is a crucial mechanism in the carcinogenesis of cervical cancer (Yim and Park, 2005; Jiang et al., 2019; Gutiérrez-Hoya and Soto-Cruz, 2020). Therefore, in subsequent studies, we investigated the potential of the HPV16 E6-binding affibody as a synergistic agent to enhance the antitumor effect of Z_HPV16E6_384, an HPV16 E7-binding affibody molecule previously reported to show promising therapeutic value in HPV16-positive tumors (Jiang et al., 2019). Both CCK-8 and plate colony formation assays showed that the combination of Z_HPV16E6_1235 and Z_HPV16E7_384 therapy was significantly superior to either modality alone at the same concentration in HPV16-positive cancer cell lines. In addition, the synergistic inhibitory effect of combination therapy with Z_HPV16E6_1235 and Z_HPV16E7_384 on HPV16-positive cell growth was further confirmed by Chou-Talalay analysis. Further mechanistic analyses demonstrated that the synergistic antiproliferative activity mainly depends on the induction of cell apoptosis and senescence but is not related to cell cycle arrest.

In summary, we successfully screened three novel affibody molecules and confirmed their high affinity and specificity for HPV16 E6 oncoprotein through SPR, indirect immunofluorescence, IHC and near-infrared small animal optical imaging *in vitro* and *in vivo*. The detailed mechanism underlying the cell penetration and binding of Z_HPV16E6_ affibodies to intracellular target *in vitro* and *in vivo* remains to be further investigated. Nevertheless, our data showed that treatment with affibody Z_HPV16E6_1235 could blocked the degradation of p53 by E6 and rescued the expression of p53, which in turn activated a robust p53-mediated transcriptional program and inhibited the proliferation of HPV16-positive cell lines. More importantly, our data indicated that the combined use of Z_HPV16E6_1235 and Z_HPV16E7_384 to simultaneously target the HPV16 E6 and E7 oncoproteins can significantly enhance antiproliferative activity by inducing increased cell apoptosis and senescence. Therefore, we envisage that Z_HPV16E6_1235 could be utilized as a promising starting point for developing rational strategies for both targeted therapy and molecular imaging in HPV16-positive patients.

## Data Availability Statement

The original contributions presented in the study are included in the article/Supplementary Material, further inquiries can be directed to the corresponding authors. In addition, DNA sequence for the three selected HPV16 E6 binding affibody molecules has been deposited in Genbank dataset (https://www.ncbi.nlm.nih.gov/genbank/), and the accession number for them is MW888864, MW888865, and MW888866, respectively.

## Ethics Statement

The animal study was reviewed and approved by the Ethical Committee of Wenzhou Medical University.

## Author Contributions

LZ: conceptualization and project administration. JZ and LZ: data curation, formal analysis, and writing-original draft. MC and LZ: funding acquisition, resources, and supervision. JZ, SK, QW, YG, QL, LW, JC, QD, WD, SC, SZ, and JC: investigation. JZ, SK, and LZ: methodology. JZ and JC: software. JZ: validation and visualization. All authors contributed to the article and approved the submitted version.

## Conflict of Interest

The authors declare that the research was conducted in the absence of any commercial or financial relationships that could be construed as a potential conflict of interest.

## Footnotes

1 www.ncbi.nlm.nih.gov

2 http://www.combosyn.com

## Supplementary Material

Supplementary Figure 1 ELISA screening for target-binding activity of potential HPV16 E6-binding affibody molecules from 66 clones.

Supplementary Figure 2 Amino acid sequence of the wild-type Z domain aligned to amino acid sequences of 42 unique affibody variants showing binding activity in ELISA. The 13 randomized amino acid positions are presented; dashes (–) indicate amino acids identical to those in the wild-type Z domain. The values on the right indicate the number of times each variant was detected out of 66 clones.

Supplementary Figure 3 *In vivo* biodistribution of affibody molecules in healthy normal mice. **(A)** The labeled affibody molecules were confirmed by SDS-PAGE and further detected at wavelengths of 730–950 nm by an *in vivo* fluorescence imaging system. **(B,C)** After tail vein injection with DyLight 755-labeled affibody molecules, fluorescence images were obtained from mice at different time points. Kidney uptake was prominent for the accumulation of affibody molecules. The accumulation of affibody molecules maximally occurred at 8 hpi and then decreased over the time course. The signal was undetectable at 72 hpi.
