# Supplementary figures and images for: Novel Affibody Molecules Targeting the HPV16 E6 Oncoprotein Inhibited the Proliferation of Cervical Cancer Cells
Source: Front Cell Dev Biol. 2021 May 24;9:677867. doi: 10.3389/fcell.2021.677867 (PMC8181454; doi:10.3389/fcell.2021.677867)

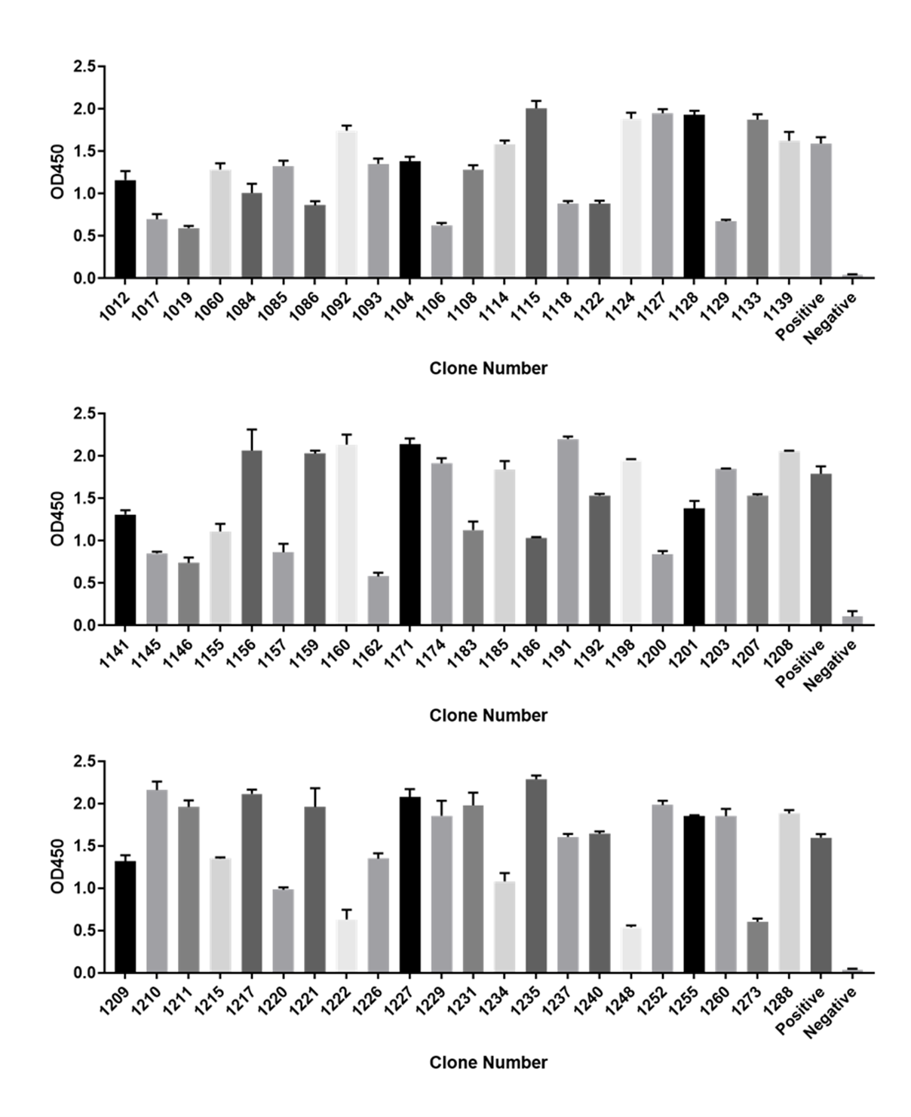

Supplement: Supplementary Figure 1 — ELISA screening for target-binding activity of potential HPV16 E6-binding affibody molecules from 66 clones. [file Image_1.TIF]

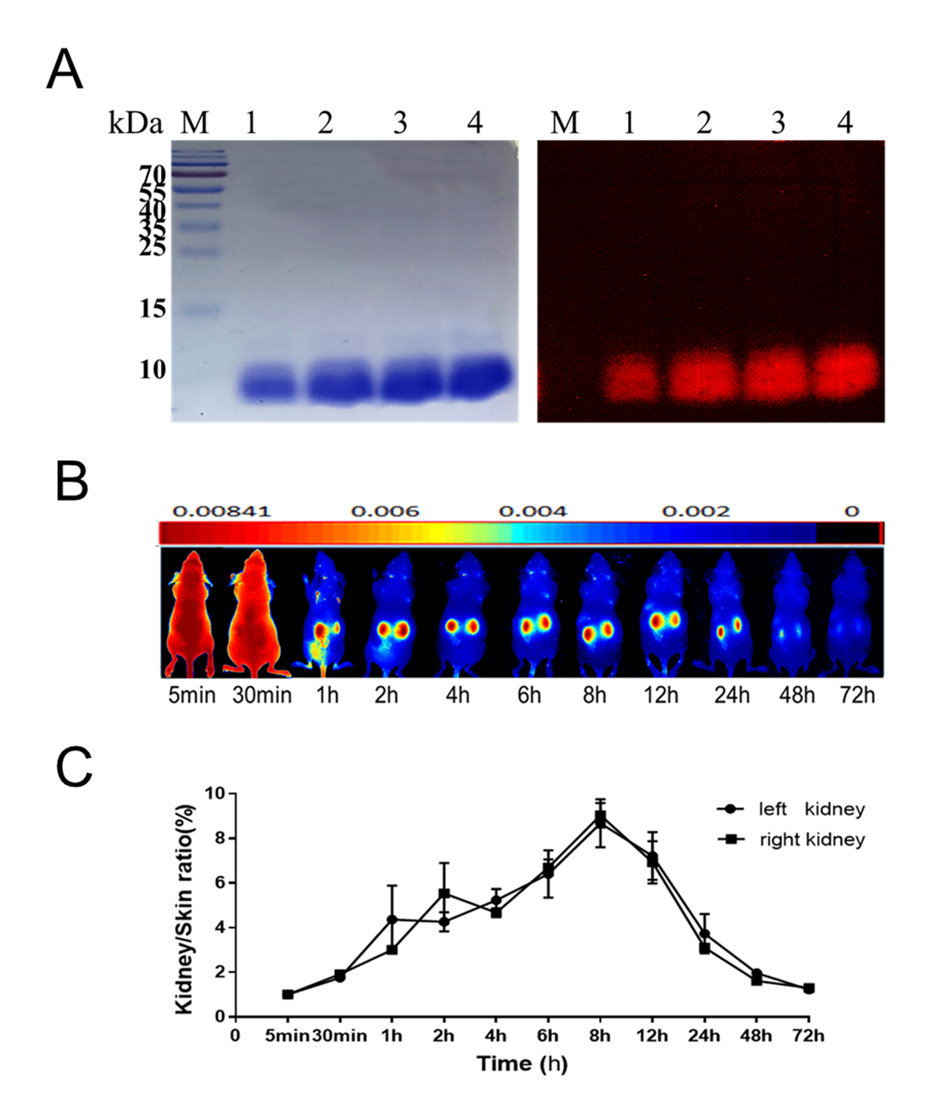

Supplement: Supplementary Figure 2 — Amino acid sequence of the wild-type Z domain aligned to amino acid sequences of 42 unique affibody variants showing binding activity in ELISA. The 13 randomized amino acid positions are presented; dashes (–) indicate amino acids identical to those in the wild-type Z domain. The values on the right indicate the number of times each variant was detected out of 66 clones. [file Image_3.TIF]

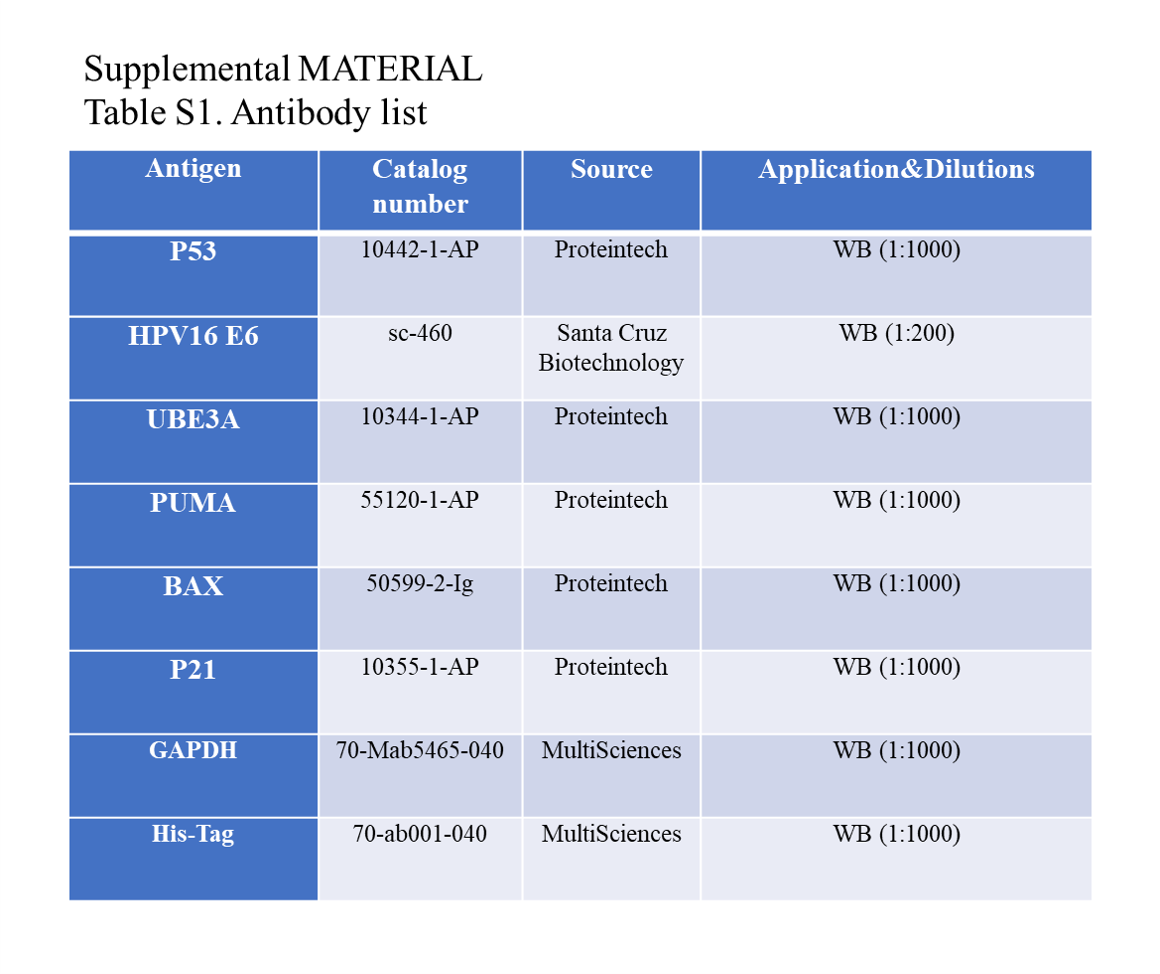

Supplement: Supplementary Figure 3 — In vivo biodistribution of affibody molecules in healthy normal mice. (A) The labeled affibody molecules were confirmed by SDS-PAGE and further detected at wavelengths of 730–950 nm by an in vivo fluorescence imaging system. (B,C) After tail vein injection with DyLight 755-labeled affibody molecules, fluorescence images were obtained from mice at different time points. Kidney uptake was prominent for the accumulation of affibody molecules. The accumulation of affibody molecules maximally occurred at 8 hpi and then decreased over the time course. The signal was undetectable at 72 hpi. [file Image_4.TIF]

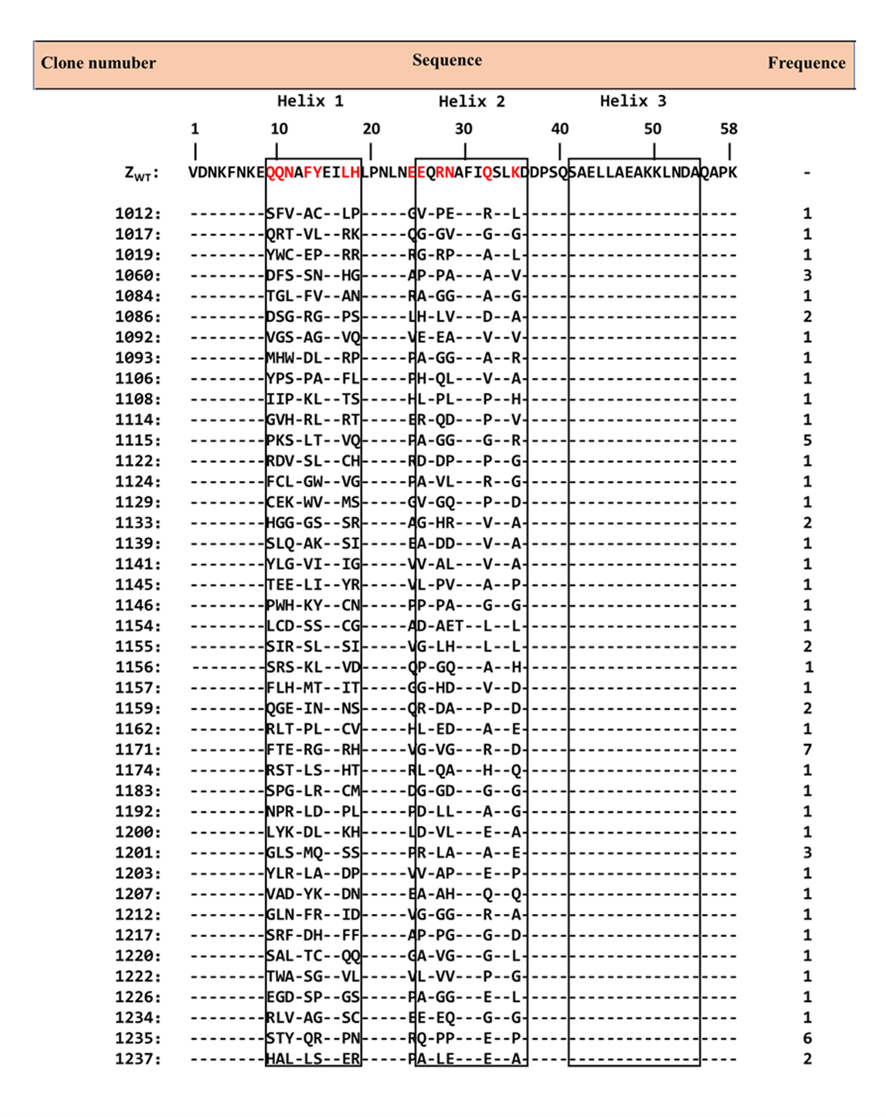

Supplement: Supplementary file 4 [file Image_2.TIF]
